# Preparing for responsive management versus preparing for renal dialysis in multimorbid older people with advanced chronic kidney disease (Prepare for Kidney Care): Study protocol for a randomised controlled trial.

**DOI:** 10.1186/s13063-024-08509-8

**Published:** 2024-10-17

**Authors:** Jo Worthington, Alexandra Soundy, Jessica Frost, Leila Rooshenas, Stephanie J. MacNeill, Alba Realpe Rojas, Kirsty Garfield, Yumeng Liu, Karen Alloway, Yoav Ben-Shlomo, Aine Burns, Joseph Chilcot, Jos Darling, Simon Davies, Ken Farrington, Andrew Gibson, Samantha Husbands, Richard Huxtable, Helen McNally, Emma Murphy, Fliss E. M. Murtagh, Hugh Rayner, Caoimhe T. Rice, Paul Roderick, Chris Salisbury, Jodi Taylor, Helen Winton, Jenny Donovan, Joanna Coast, J Athene Lane, Fergus J. Caskey

**Affiliations:** 1 Bristol Trials Centre, Bristol Medical School, University of Bristol, 1–5 Whiteladies Road, Bristol, BS8 1NU UK; 2 Population Health Sciences, Bristol Medical School, University of Bristol, Canynge Hall, 39 Whatley Road, Bristol, BS8 2PS UK; 3 North Bristol NHS Trust, Southmead Hospital, Bristol, BS10 5NB UK; 4 UCL Department of Nephrology, Royal Free Hospital, University College, London, NW3 2QG UK; 5 Institute of Psychiatry, Psychology & Neuroscience, King’s College London, London, WC2R 2LS UK; 6 Public and Patient Involvement Representative, London, UK; 7 Institute for Science and Technology in Medicine, Keele University, Keele, ST5 5BG UK; 8 Renal Unit, East and North Hertfordshire NHS Trust, Coreys Mill Lane, Stevenage, SG1 4AB UK; 9 ARC West, Whitefriars, Lewins Mead, Bristol, BS1 2NT UK; 10 Centre for Care Excellence, Coventry University and University Hospitals Coventry and Warwickshire NHS Trust, Coventry, UK; 11 Institute for Cardio-Metabolic Medicine, University Hospitals Coventry and Warwickshire NHS Trust, Coventry, UK; 12 Wolfson Palliative Care Research Centre, Hull York Medical School, University of Hull, Hull, HU6 7RX UK; 13 Renal Unit, Birmingham Heartlands Hospital, Bordesley Green E, Birmingham, B9 5SS UK; 14 Faculty of Medicine, University of Southampton, University Road, Southampton, SO17 1BJ UK

**Keywords:** Chronic kidney disease, Kidney failure, Dialysis, Conservative kidney management, Supportive care, Palliative care, Advance care planning, Randomised controlled trial, Integrated qualitative research, Quality-adjusted life years

## Abstract

**Background:**

Chronic kidney disease (CKD) prevalence is steadily increasing, in part due to increased multimorbidity in our aging global population. When progression to kidney failure cannot be avoided, people need unbiased information to inform decisions about whether to start dialysis, if or when indicated, or continue with holistic person-centred care without dialysis (conservative kidney management). Comparisons suggest that while there may be some survival benefit from dialysis over conservative kidney management, in people aged 80 years and over, or with multiple health problems or frailty, this may be at the expense of quality of life, hospitalisations, symptom burden and preferred place of death. Prepare for Kidney Care aims to compare preparation for a renal dialysis pathway with preparation for a conservative kidney management pathway, in relation to quantity and quality of life in multimorbid, frail, older people with advanced CKD.

**Methods:**

This is a two-arm, superiority, parallel group, non-blinded, individual-level, multi-centre, pragmatic trial, set in United Kingdom National Health Service (NHS) kidney units. Patients with advanced CKD (estimated glomerular filtration rate < 15 mL/min/1.73 m^2^, not due to acute kidney injury) who are (a) 80 years of age and over regardless of frailty or multimorbidity, or (b) 65–79 years of age if they are frail or multimorbid, are randomised 1:1 to ‘prepare for responsive management’, a protocolised form of conservative kidney management, or ‘prepare for renal dialysis’. An integrated QuinteT Recruitment Intervention is included. The primary outcome is mean total number of quality-adjusted life years during an average follow-up of 3 years. The primary analysis is a modified intention-to-treat including all participants contributing at least one quality of life measurement. Secondary outcomes include survival, patient-reported outcomes, physical functioning, relative/carer reported outcomes and qualitative assessments of treatment arm acceptability. Cost-effectiveness is estimated from (i) NHS and personal social services and (ii) societal perspectives.

**Discussion:**

This randomised study is designed to provide high-quality evidence for frail, multimorbid, older patients with advanced CKD choosing between preparing for dialysis or conservative kidney management, and healthcare professionals and policy makers planning the related services.

**Trial registration:**

ISRCTN, ISRCTN17133653 (https://doi.org/10.1186/ISRCTN17133653). Registered 31 May 2017.

**Supplementary Information:**

The online version contains supplementary material available at 10.1186/s13063-024-08509-8.

## Administrative information

Note: the numbers in curly brackets in this protocol refer to SPIRIT checklist item numbers. The order of the items has been modified to group similar items (see http://www.equator-network.org/reporting-guidelines/spirit-2013-statement-defining-standard-protocol-items-for-clinical-trials/).

**Table:** 

Title {1}	Preparing for responsive management versus preparing for renal dialysis in multimorbid older people with advanced kidney disease (Prepare for Kidney Care): study protocol for a randomised controlled trial.
Trial registration {2a and 2b}.	ISRCTN 17133653. Prospectively registered 31 May 2017.
Protocol version {3}	26th June 2024, version 10.0
Funding {4}	National Institute for Health and Care Research (NIHR) Health Technology Assessment (HTA) Programme (15/57/39)
Author details {5a}	1 Bristol Trials Centre, 1–5 Whiteladies Road, Bristol Medical School, University of Bristol, Bristol, BS8 1NU, UK 2 Population Health Sciences, Bristol Medical School, University of Bristol, Canynge Hall, 39 Whatley Road, Bristol, BS8 2PS, UK 3 North Bristol NHS Trust, Southmead Hospital, Bristol, BS10 5NB, UK 4 UCL Department of Nephrology, Royal Free Hospital, University College London, NW3 2QG, UK 5 Institute of Psychiatry, Psychology & Neuroscience, King’s College London, WC2R 2LS, UK 6 Public and Patient Involvement representative, UK 7 Institute for Science and Technology in Medicine, Keele University, ST5 5BG, UK. 8 Renal Unit, East and North Hertfordshire NHS Trust, Coreys Mill Lane, Stevenage, SG1 4AB, UK 9 ARC West, Whitefriars, Lewins Mead, Bristol, BS1 2NT, UK 10 Centre for Care Excellence, Coventry University and University Hospitals Coventry and Warwickshire NHS Trust, Coventry, UK 11 Institute for Cardio-Metabolic Medicine, University Hospitals Coventry and Warwickshire NHS Trust, Coventry, UK 12 Wolfson Palliative Care Research Centre, Hull York Medical School, University of Hull, HU6 7RX, UK 13 Renal Unit, Birmingham Heartlands Hospital, Bordesley Green E, Birmingham, B9 5SS, UK 14 Faculty of Medicine, University of Southampton, University Road Southampton, SO17 1BJ, UK
Name and contact information for the trial sponsor {5b}	North Bristol NHS Trust, Southmead Hospital, Bristol, BS10 5NB, UK. researchsponsor@nbt.nhs.uk
Role of sponsor {5c}	The sponsor played no part in study design and will play no part in the collection, management, analysis, and interpretation of data. The sponsor is, however, responsible for overall oversight of the trial. Drafts of all reports will be shared with the Sponsor for approval prior to submission for publication.

## Introduction

### Background and rationale {6a}

Every year in the United Kingdom (UK) more than 3500 people aged 65 and over develop symptomatic kidney failure and start dialysis, and this number continues to rise [1, 2]. Transplantation is not an option for most of these people, and while dialysis extends life for some, the associated survival and quality of life (QoL) benefits are uncertain for older people with multiple health problems or frailty. Dialysis has a considerable impact on daily life [3]. Of patients aged 65 and over commencing dialysis, 86% receive haemodialysis (typically three times a week for 4 h, at a hospital or clinic) and 14% receive peritoneal dialysis (typically four times a day for 30 min, or overnight by machine, at home) [1]. Preparation for this must start months in advance, as patients choose a treatment option and may need to undergo any necessary surgery for dialysis access. As they develop symptoms and start dialysis at different levels of kidney function, knowing when to begin this preparation is difficult. Although prognosis on dialysis is well documented (registry data show 3-year survival to be 55% in people aged 65–74 years and 40% in people aged 75 years and over [4]), it is not known how long the same individuals would have lived with conservative kidney management.

Some patients aged 65 years and over choose not to prepare for dialysis and instead have conservative kidney management, comprising care without preparing for dialysis [5], but the content and availability of conservative kidney management services has been shown to vary widely in the UK [6].

Evidence of the comparative effectiveness of these approaches—dialysis and conservative kidney management—is entirely observational and so prone to confounding by indication bias (if all indications for treatment initiation are not captured) and survival bias (if the time point equivalent to starting dialysis is not known in the conservative kidney management group) [7]. What evidence there is suggests modest, and not always consistent, survival advantages from having dialysis, in some patient groups—those aged 70 and over with a WHO performance status of 3 and above [8], those aged 75 and over with two or more comorbidities [9], and those aged 80 and over regardless of comorbidity [8]. Considering QoL and secondary outcomes such as hospitalisations, symptom burden and preferred place of death, however, evidence suggests small, and again not always consistent, advantages from conservative kidney management [7]. In patients initiating dialysis, there can be an improvement in some domains, but a decline in others—Satisfaction with Life Scale [10], and the Kidney Disease Quality of Life (KDQoL) parameters Effect of Kidney Disease and Burden of Kidney Disease [11]. QoL is also significantly affected in carers of people on dialysis [12] and the experience of caring for people receiving conservative kidney management presents substantial difficulties [13].

The uncertainty about the relative benefits of dialysis and conservative kidney management is reflected in the variations in practice. A 2013 survey of UK kidney units showed great variability in the proportion of patients aged 75 and over choosing conservative kidney management depending on which kidney unit they attended [6]. This variation was too large to be attributed to patient factors alone, with contextual factors, such as differing healthcare professional interpretation of the observational data, likely to be contributing.

With the size of the UK population aged 65 and over predicted to increase by 60% (from 10.3 to 16.9 m) by 2035 [14], and healthcare costs predicted to continue to increase [15], the optimal management of frail, multimorbid older people with advanced chronic kidney disease (CKD) will remain highly relevant to national health services. The importance of end of life kidney care in the UK was recognised in 2005 in Part 2 of the National Service Framework [16]. More recently it has been highlighted globally by Kidney Disease Improving Global Outcomes [5] and the International Society of Nephrology [17, 18] who have held consensus meetings to coordinate efforts in this area. Given the human and economic impact of dialysis in older people, there is a pressing need to establish the comparative effectiveness of preparing for conservative kidney management or preparing for dialysis, and to provide high-quality, unbiased evidence to inform frail, multimorbid, older patients with advanced CKD, their relatives and their clinical teams when making extremely difficult decisions about whether to follow a ‘prepare for dialysis’ or a ‘prepare for conservative kidney management’ pathway. The Prepare for Kidney Care randomised controlled trial (RCT) is the first study that seeks to generate this evidence, comparing preparing for renal dialysis with preparing for an optimised form of conservative kidney management, developed with experts and patients specifically for the study (‘responsive management’, see section “Intervention description {11a}”).

### Objectives {7}

#### Primary objective

To determine the relative effectiveness of preparing for responsive management versus preparing for renal dialysis on quality-adjusted life years (QALYs) over an estimated average follow-up of 3 years in an individual-level, pragmatic RCT in frail, multimorbid, older people with advanced CKD.

#### Secondary objectives

1. To determine the effect of the intervention on:
  i. Survival.
  ii. Hospital-free days.
  iii. Physical functioning: timed get up and go and hand grip strength.
  iv. Patient reported outcomes: generic and disease-specific QoL, capability gain, patient treatment burden and impact on carers.
  v. Cost-effectiveness: Incremental cost-per QALY gained from (i) an NHS and personal social services (PSS) perspective (primary economic analysis) and (ii) a societal perspective, and incremental cost per years of full/sufficient capability equivalence gained.
2. To fully understand the external validity of the trial through observational cohorts with linkage to routine NHS data.
3. To explore patients’, relatives’ and health care professionals’ perspectives on the acceptability of preparation for dialysis and preparation for responsive management.

### Trial design {8}

**Fig. 1:**
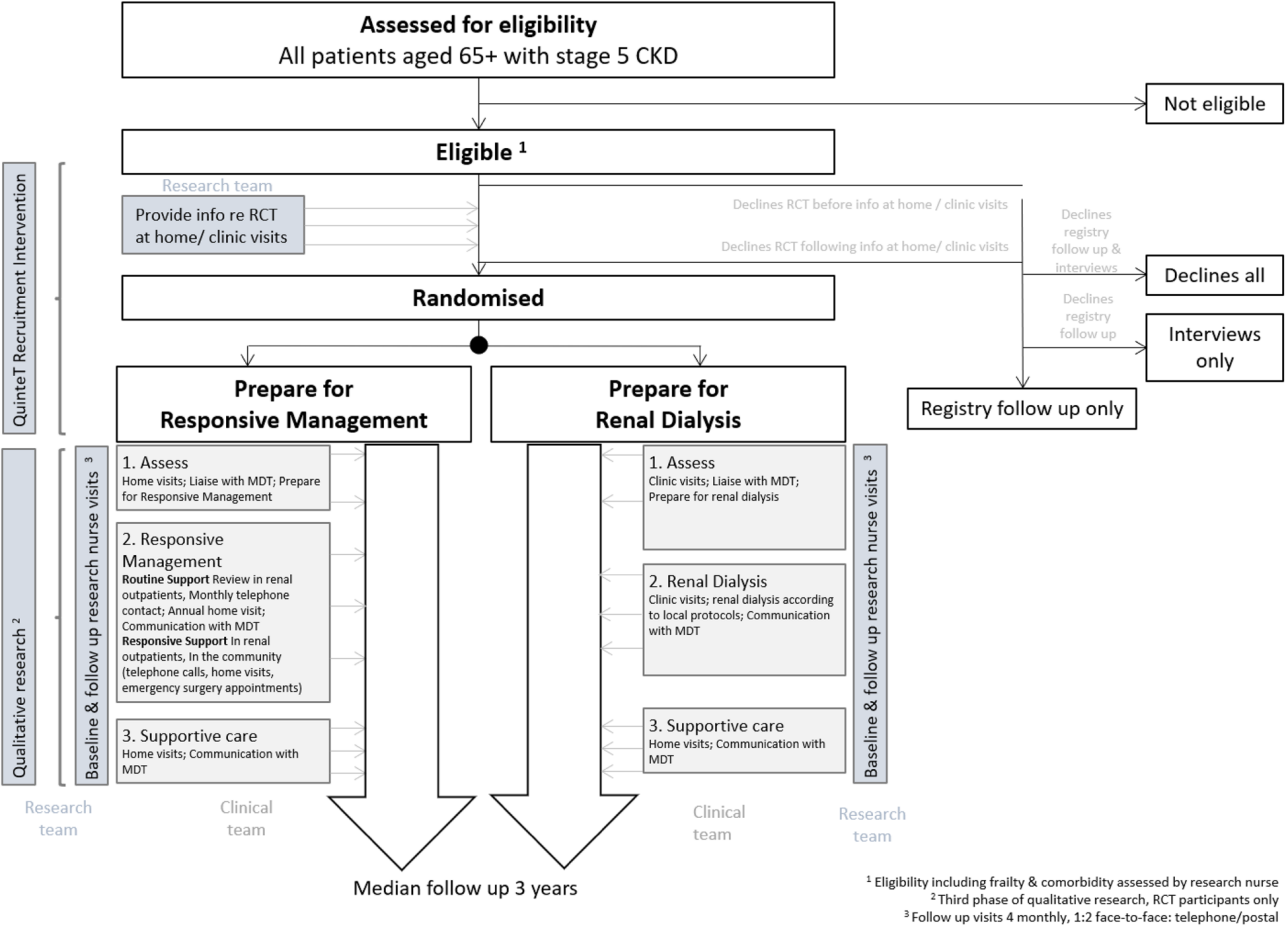
Trial flow diagram

The study is a two-arm, superiority, parallel group, non-blinded, individual-level, pragmatic RCT in frail, multimorbid, older people with advanced CKD. Participants are randomised 1:1 to prepare for responsive management or prepare for renal dialysis. The trial design included an internal pilot recruitment phase of 6 months’ duration, primarily to verify that recruitment was possible before progression to the main phase of the trial. An integrated ‘QuinteT Recruitment Intervention’ (QRI) is included to identify and overcome recruitment issues. The trial flow diagram is provided in Fig. 1.

#### Observational cohorts

The generalisability of the RCT findings will be explored after the main results have been published by studying two external, parallel, observational cohorts: (1) individuals eligible for the RCT who decline randomisation but consent to baseline data collection and follow-up through linkage to routine healthcare data and central administration of quality of life questionnaires (the registry follow up study, RFU) and (2) individuals meeting the eligibility criteria in routine NHS databases who may or may not have been invited to participate in the RCT or observational study (Additional file 1).

#### The QuinteT Recruitment Intervention and integrated qualitative research

A programme of qualitative research and the QRI are embedded throughout the RCT:

- Stage 1, conducted at the pre-trial stage, is complete. It comprised interviews with healthcare professionals (HCPs) to investigate current approaches to managing the eligible patient population and their perspectives on how the proposed trial design/protocol could be integrated with their routine practices. Findings from this work, published separately [19], refined the design and protocol for the main RCT outlined in this paper, including changes to the intervention’s name (from ‘prepare for conservative care’ to ‘prepare for responsive management’) and refinements to the intervention’s components.
- Stage 2 comprises application of the QRI to understand and address recruitment challenges as the RCT is underway [20]. Details of the QRI are reported under ‘recruitment’, below.
- Stage 3 seeks to understand acceptability of the trial’s treatment arms, trial processes and reasons for non-compliance, from patients’, relatives’ and HCPs’ perspectives. Further details are available in additional file 2.

## Methods: participants, interventions and outcomes

### Study setting {9}

This is a national, multi-centre trial, recruiting patients from UK secondary care kidney units, with the intervention being delivered across primary care, secondary care and the community. Thirty one adult kidney units providing dialysis services in England, Scotland, Wales and Northern Ireland have been recruited. A full list of study sites is available on the trial website [21].

### Eligibility criteria {10}

Specialist kidney doctors, specialist kidney nurses and research nurses in each centre identify patients known to kidney services and considering their treatment options.

#### Inclusion criteria

Patients meeting inclusion criteria are known to renal services with new or existing stage 5 CKD (defined as an estimated glomerular filtration rate (eGFR) less than 15 mL/min/1.73 m^2^ that is not considered to be due to acute kidney injury by the clinical team, and with at least one confirmatory reading less than 15 mL/min/1.73 m^2^ in the preceding 12 months) and either:

- Aged 65 years and over with a World Health Organization (WHO) performance status of 3 or above (i.e. 3 = symptomatic and in a chair or in bed for greater than 50% of the day, but not bedridden; 4 = completely disabled; cannot carry out any self-care; totally confined to a bed or chair), as assessed during the screening period [8], or
- Aged 65 years and over with a comorbidity score of 2 or more [22] or
- Aged 80 years and over [8].

#### Exclusion criteria

- Unable to consent, e.g. significant cognitive impairment or psychiatric disorder
- Not medically fit for dialysis
- Within 4 weeks of needing to start dialysis
- Previous kidney transplant
- Active on the kidney transplant waiting list or being worked up for the kidney transplant waiting list

#### Inclusion criteria for family members, friends and carers

With the patient’s consent, family members, friends and carers will be recruited to the carer elements of the study if they are:

- Aged 18 years and over, and
- Identified by the patient as the person closest to them, usually a family member, close friend, informal caregiver or neighbour, and
- Able to give informed consent and to complete the carer questionnaires.

### Who will take informed consent? {26a}

All patients/carers joining the RCT provide written, informed consent to participate. Consent is obtained by GCP-trained doctors, research nurses or delegated team members. Up to three initial contacts can be conducted to ensure the patient and family are fully informed before consent.

### Additional consent provisions for collection and use of participant data and biological specimens {26b}

#### Registry follow-up study (RFU)

Eligible patients who decline participation in the RCT are invited to consent to participate in a cohort study (additional file 1). All patients joining the RFU provide informed consent, obtained as per the RCT.

#### Data linkage

The lawful bases for the processing of data for the purposes of linkage to other routine sources are set out in the privacy notice on the trial website [21].

#### Integrated QRI and qualitative research

Written or verbal informed consent is obtained from all participants following receipt of relevant study information sheets for the QRI and qualitative aspects of the study. Potential participants include patients, trial personnel and HCPs in recruiting sites. Dedicated information sheets and consent forms are available for each element of the study and each group approached.

### Interventions

#### Explanation for the choice of comparators {6b}

The comparators—prepare for renal dialysis and prepare for responsive management—are based on the two treatment pathways currently available to frail, multimorbid, older people with advanced CKD, not suitable for kidney transplantation, in the UK. As outlined in the “Background and Rationale {6a}”, systematic reviews of the available observational evidence have suggested modest, and not always consistent, survival advantages of preparing for renal dialysis in some patient groups [7]—those aged 70 years and over with a WHO performance status of 3 and above [8], those aged 75 years and over with two or more comorbidities [9] and those aged 80 years and over regardless of comorbidity [8]. Considering QoL and secondary outcomes such as hospitalisation, symptom burden and place of death, however, evidence suggests small, and not always consistent, advantages from conservative kidney management [7]. The significant variation in reported rates of dialysis and conservative kidney management between UK kidney units suggests lack of certainty regarding superiority of one pathway versus the other, i.e. community equipoise.

#### Intervention description {11a}

The intervention arm—‘prepare for responsive management’—is an optimised form of conservative kidney management based on international guidance [5], and developed by UK expert consensus with PPI involvement, and informed by the pre-trial qualitative findings [19]. The difference between the two pathways is that patients randomised to prepare for responsive management have regular support from the kidney unit staff (assessment of their symptoms and advance care planning that includes consideration of priorities for care at home visits and by telephone) and support that responds to their needs (from kidney unit staff, palliative care teams and community staff). They avoid the scans and surgery that normally take place when patients prepare for renal dialysis and therefore any complications of that access surgery. The aim of the pathway is to support patients and respond to their needs in their preferred place of care so that they do not feel they have to swap to dialysis to feel safe and be kept comfortable if/when they develop kidney failure.

#### Prepare for responsive management—the intervention

Following allocation of the participant to the prepare for responsive management arm, the research nurse informs the patient and their family/ friend/ carer about what will happen in terms of (a) responsive management visits/ contacts as outlined in the study ‘Clinical Care Manual’ and (b) research team visits.

The intervention is delivered by HCPs who are expert in the assessment and management of people with advanced CKD. The clinical care can be considered in three stages; assess, responsive management and supportive care, as detailed below.

I. *Assess*—a comprehensive care assessment is carried out in the patient’s home to include the following, as appropriate:
  Completion of symptom checklist (IPOS-renal or equivalent)
  Symptom control and management
  Continuity and coordination of care, access to services
  Psychosocial needs
  Information / communication needs
  Advance care planning
  Assessment of caregiver
II. *Responsive management—*routine support and responsive support
  Routine support
    Monthly telephone contacts (to occur once in each calendar month in which there is not a clinic or home visit) to include an abridged care assessment.
    Annual home visits from the healthcare provider delivering responsive management to reassess the patient’s needs (as per ‘Assess’) and priorities for care in their environment. The visit and plan is discussed and agreed with the multidisciplinary team. If, despite optimisation of their medication, the patient’s symptoms are proving difficult to adequately control and the patient still wishes to follow a responsive management pathway, treatment will escalate to ‘Supportive care’.
    Clinic visits with the patient’s consultant nephrologist as per routine practice, but with one of these visits being replaced with the annual home visit. The content of these clinic visits should not include preparation for dialysis unless the patient decides to withdraw from responsive management.
  Responsive support
    In response to each individual patient’s evolving personal, social and clinical situation, a range of options are available to ensure that they feel as supported and safe as possible in their preferred place of care. The option chosen will depend on the individual scenario and the clinical assessment of the most locally appropriate way to respond, but could include:
    Supporting the patient at home: telephone calls from the kidney unit team, community team, or general practitioner (GP); appointments at the GP surgery; home visits from the kidney unit team, community team, or GP.
    Supporting the patient at the hospital/ clinic: kidney outpatient clinic visits; palliative care outpatient clinic visits; admission to hospital or the hospice, if required.
   Symptoms, results or events that should trigger senior clinical review: Although all clinical teams will have their own local arrangements for seeking senior clinical review of a patient about whom they have concerns, it is necessary for the safe delivery of the intervention to agree a minimum threshold across all sites for making the patient’s nephrologist aware of any change in the status of their kidney condition. These triggers fall into a number of categories.
    Symptoms
    Any severe or overwhelming symptoms on the IPOS-renal or equivalent reported for the first time during planned or unplanned telephone or face-to-face healthcare professional contact
  Blood results
    An eGFR below 6 mL/min/1.73 m^2^
    A potassium above 6.5 mmol/L
    A urea above 40 mmol/L
  Events
    An unplanned GP/emergency department/hospital attendance due to fluid overload
    An unplanned GP/emergency department/hospital attendance due to infection causing systemic illnesses
  Other
    Concern about unexplained or rapid loss of flesh weight (i.e. after allowing for changes in weight due to removal of fluid)
    The patient, or their family/ friend/ carer, is expressing doubts about the appropriateness of continuing on the allocated treatment that cannot be resolved
    The HCP believes that a senior clinical review is required
III. *Supportive care*

If the patient develops symptoms of kidney failure that cannot be adequately controlled, they should progress to the Supportive care stage. This transition may occur at a monthly telephone contact or face-to-face visit or between contacts, for example during an inter-current illness.

The advance care plan and any other documentation relating to priorities for care must be reviewed and the appropriate local community and palliative care services activated and coordinated to achieve good end-of-life care. This must be done rapidly to prevent inappropriate default cure-centred care being administered in the event of a sudden deterioration.

In principle, the package of care should aim to deliver the following five patient priorities for quality end-of-life care [23]:

- Receiving adequate pain and symptom management
- Avoiding inappropriate prolongation of dying
- Achieving a sense of control
- Relieving burden on loved ones
- Strengthening relationships with loved ones

The kidney unit team may hand over day-to-day management to the local community teams—palliative care and general practice.

### Prepare for renal dialysis—the comparator

Prepare for renal dialysis is the comparator treatment in the trial and intended to represent routine care in the NHS for someone who intends to start dialysis if/ when they and their clinical team decide it is appropriate. The clinical care can be considered in three stages; assess, renal dialysis and supportive care. The Assess stage can include provision of information about the different dialysis options, preparation for these (vascular access scans or surgical appointments to plan Tenchkoff catheter insertion, surgery to create permanent dialysis access and/or procedures to insert temporary dialysis access), psychology support, dietary advice, advance care planning and home visits, as considered appropriate. If the patient’s kidney function declines, they decide, with their clinical team, when and how to initiate their chosen modality of dialysis and move to the renal dialysis stage. If external things change in their life, or their symptoms and quality of life are not acceptable on dialysis, patients can choose to discontinue dialysis treatment at any point and move to the Supportive care stage.

#### Criteria for discontinuing or modifying allocated interventions {11b}

Participants can discontinue (a) allocated trial treatment or (b) providing data to the trial, at any time. In both cases all efforts ethically appropriate will be made to report the reason for discontinuation as thoroughly as possible on the withdrawal form.

Should a participant wish to discontinue allocated trial treatment, efforts will be made to continue to obtain follow-up data, with the permission of the patient or family/ friend/ carer, as appropriate. They may also be invited to take part in a qualitative interview, as part of the investigation of acceptability of the trial treatment arms/processes. On discontinuation of allocated treatment, the participant will return to usual care as deemed appropriate by their treating clinical team.

#### Strategies to improve adherence to interventions {11c}

Process information is documented in the electronic Clinical Team Activity Record (CTAR). The CTAR is updated every time there is contact with a patient or carer or HCP. The research nurse uses the CTAR alongside the clinical notes to capture delivery of relevant components of care to patients in both arms of the trial in the follow-up case report form (CRF). Compliance is also monitored through regular site compliance surveys. Identified compliance issues are addressed with sites and additional training provided, as required.

The primary analysis is intention-to-treat. For this reason, even if a patient is documented as deviating from protocol or withdrawing from their randomised treatment, they will be encouraged to continue with study visits/contacts and patient questionnaires.

#### Relevant concomitant care permitted or prohibited during the trial {11d}

Participants in both arms can receive any routine NHS care considered appropriate by their treating clinical team, with the following exceptions:

- Participants randomised to prepare for responsive management will be considered to have discontinued allocated treatment if they make a decision, recorded in their medical records, with their clinical team to make plans to prepare for renal dialysis, or have a dialysis access procedure (surgery or line insertion) for kidney failure, or start dialysis for kidney failure.
- Participants randomised to prepare for renal dialysis will be considered to have discontinued their allocated treatment if they make a decision, with their clinical team, recorded in their medical records and in a letter to the GP, not to start dialysis in the future, even if they become symptomatic of kidney failure.

#### Provisions for post-trial care {30}

Following the end of the trial, continued provision of conservative kidney management in the format specified for the trial (i.e. responsive management) will be at the discretion of the treating clinical team and is likely to depend on the trial results. Participants will be informed of this in the written information given to them when they are considering entering the trial.

#### Outcomes {12}

##### Primary outcome measure

The primary outcome is the mean total number of QALYs observed in the two arms between first patient recruited and the end of data collection (31st August 2025), using the EQ-5D-5L measured 4 monthly during this period to derive the health utility value with appropriate imputation methods for missing values and the area under the curve approach. After death, patients are allocated a utility value of 0 therefore continue to contribute data to the study regardless of survival. Based on projected recruitment patterns, we estimate we will have, on average, 3 years of data on patients from which to calculate QALYs (i.e. on average at least 3 years of data from first patient recruited to end of data collection).

This outcome has been chosen after extensive discussion with patients and patient groups who were very clear that they care about both quality of life and quantity of life, and evidence that patients may be willing to forgo some quantity of life for better quality of life [24].

##### Secondary outcome measures

Survival outcomes (from baseline to the end of follow-up):

- All-cause mortality
- Cause-specific mortality
- Place of death
- Hospital-free days

Patient-reported outcomes:

- Generic quality of life using the EuroQol 5-dimension 5-level (EQ-5D-5L) [25]
- Disease-specific symptom burden using the integrated palliative outcome scale-renal (IPOS-renal) [26]
- Capability gain specific to older persons using the ICEpop CAPability (ICECAP-O) [27]
- Capability during end-of-life care using the ICECAP-SCM [28]
- Treatment burden using the multimorbidity treatment burden questionnaire (MTBQ) [29]

Physical functioning outcomes:

- Timed get up and go [30]—summary score at 12 monthly time points and changes over time
- Grip strength (Jamar hand dynamometer) [31]—summary score at 12 monthly time points and changes over time

Relative/carer-reported outcomes:

- Impact on carers using the PAlliative Care in chronic Kidney diSease impact on carers questionnaire (PACKS) [32]
- QUALYCARE post-bereavement survey obtaining retrospective information covering the 1 week preceding death, if the patient dies [33].

Health economic outcomes:

- Incremental cost-per QALY gained from a health and social care provider perspective and a secondary societal perspective including patients and close persons
- Cost per year of full/sufficient capability equivalence gained, from a health and social care provider perspective and secondary societal perspective including patients and close persons.

Patient, relative and HCP-reported acceptability of the treatment arms (particularly, the intervention):

- Captured through qualitative interviews (see additional file 2).

#### Participant timeline {13}

**Fig. 2:**
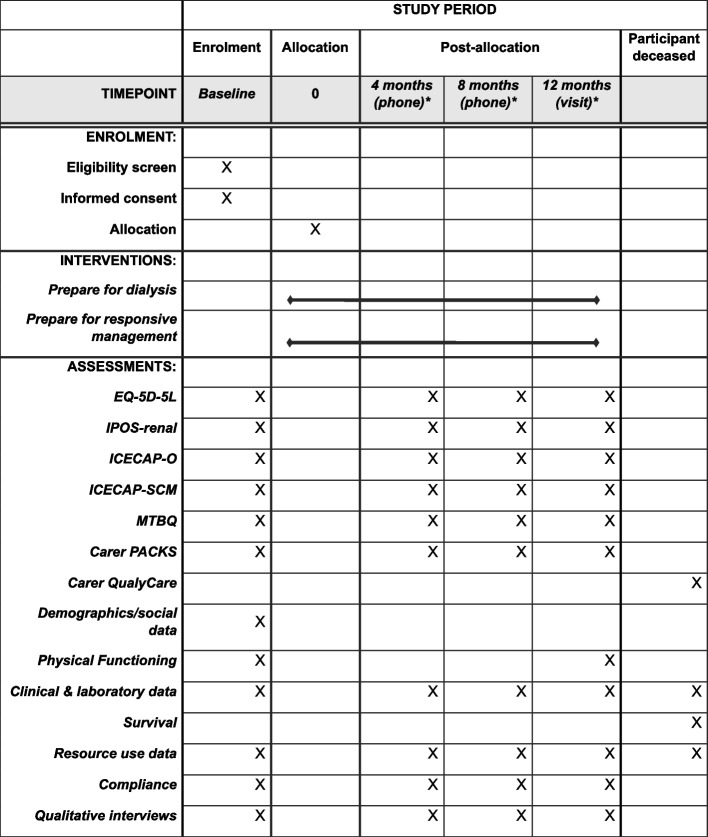
Schedule of enrolment, interventions and assessments *Repeated until the end of the follow up period (31 August 2025)

The time schedule of enrolment, interventions and assessments are shown in a schematic diagram (Fig. 2).

#### Sample size {14}

A sample size of 512 has 90% power to detect a difference between the pathways of 0.345 QALYs (5% two-sided) over 3 years of follow-up based on EQ-5D-5L. A discrete choice experiment in 8 kidney units in Australia suggests that patients with kidney failure are willing to forgo 7 months of life expectancy to reduce the number of visits to hospital for dialysis by one per week [24]. Responsive management reduces visits to hospital. Based on a median EQ-5D utility value for being alive on dialysis of 0.69 [34], 7 months of life at this value would equate to 0.4 QALYs. We wanted to be able to detect a slightly smaller difference than this of 6 months between the two pathways, equivalent to 0.345 QALYs.

For the purposes of informing our calculation, we examined data from a longitudinal study [35] of patients with CKD where utility score data were available at multiple points during follow-up. In that particular study, patients were followed up from eGFR 20–30 ml/min/1.73 m^2^ until start of dialysis or end of follow-up. From these data, we calculated total QALYs over 3 years of follow-up (based on EQ-5D).

The data suggested a bimodal distribution (means 0.36 and 2.4). While we cannot exclude the possibility that a larger sample would have yielded a unimodal distribution, bi-modal distributions of EQ-5D values (which are used to calculate QALYs) have been noted in a variety of conditions including rheumatoid arthritis [36, 37] and cancer [38]. Explanations for such distributions include the algorithm used to calculate the EQ-5D score itself which tends to cluster scores in the extremity close to 1.0 (perfect health) and near 0.45 (representing moderate health) [39].

Based on summary statistics from the observed data and visual inspection of the histogram, we simulated datasets of 1,000,000 observations for comparator groups using the SKBIM command in STATA [40]. This command allows the user to simulate a bimodal distribution while specifying different characteristics of the distribution including means, standard deviations, skewness and kurtosis (for the comparator group the simulated dataset was generated with this command: skbim (0.5 0.359 0.096 2.413 2.413 0.154 1 1000000 123 0.804 2.492 0 3)). To simulate the distribution of QALYs in the intervention group, we assumed the same distribution as the comparator group, but shifted the two means by 0.345 QALYs to reflect a clinically meaningful difference we wanted to be able to detect. Simulated values greater than 3 were discarded.

Sample size calculations involved re-sampling (1000 iterations) with varying sample sizes from these simulated distributions of continuous QALY scores and performing Mann Whitney tests. To allow for possible cross over, we selected our intervention group samples by taking 95% of our sample from the intervention distribution and 5% from the comparator distribution. The same process was used to identify the comparator group sample.

Having 230 patients per group allows detection of a difference between the pathways of 0.345 QALYs with 90% power (2-sided a = 0.05). Death is not a censoring event (utility becomes and remains 0) so loss-to-follow-up should not exceed 10%. Allowing for loss-to-follow-up increased the total sample size to 512.

Following the COVID-19 pandemic, recruitment rates have improved but are still generally tracking below predicted. Following discussions with the Trial Steering Committee (TSC), Patient Advisory Group (PAG) and funder, the Trial Management Group (TMG) has agreed to extend recruitment to ensure 85% power is achieved. Using the original sample size simulations and associated assumptions, this reduces the target sample size from 512 to 446 participants.

#### Recruitment {15}

Patients referred with advanced CKD and receiving education regarding potential treatment options are identified at time of receipt of referral letter, during patients’ clinical appointments, at low-clearance or pre-dialysis clinics, or by searching kidney clinic records. Eligible patients are provided with a study invitation letter and participant information sheet. Patients ineligible due to an eGFR greater than 15 mL/min/1.73 m^2^ can be given a pre-eligibility patient information sheet as part of their kidney education package to introduce the concept of study participation as a future option.

##### Application of the QRI to optimise recruitment

Previous studies in this area have highlighted the need for RCT evidence, but also documented doubts about the viability of such a trial due to anticipated recruitment challenges [6]. In light of these concerns and previous under-recruiting trials in this field [41], we incorporated the QRI to support recruitment to Prepare for Kidney Care. The QRI is a complex intervention, designed to run concurrent to an RCT’s recruitment period, with the aim of identifying and addressing factors that compromise recruitment and informed consent [20]. The QRI’s methods were conceived during the NIHR-funded ProtecT study [42] and refined through application to over 50 other RCTs, following evidence of its success at diagnosing and addressing recruitment issues in challenging trials [20, 43, 44]. The methods underpinning the QRI have been protocolised and are reported elsewhere [20]. In brief, the QRI proceeds via two cyclical phases: phase 1 seeks to rapidly unearth factors that shape recruitment success to the particular RCT by triangulating data from a range of sources: (i) analysis of screening log information, capturing data on numbers of patients screened, eligible, approached and randomised (‘SEAR’) and reasons why patients do not proceed through these stages [45]; (ii) audio-recordings of recruitment discussions where the trial is presented to potential participants and their families/carers; (iii) interviews with trial/site staff and patients and (iv) analysis of trial documentation [46]. Phase 2 draws on these findings to design and deliver tailored ‘actions’ to improve recruitment as the trial is underway. Prepare for Kidney Care is the first renal trial to employ the QRI. Further details of application of QRI methods to Prepare for Kidney Care are available in additional file 3.

### Assignment of interventions: allocation

#### Sequence generation {16a}

Randomisation allocations were generated by an automated web/telephone randomisation system provided by the Bristol Trials Centre until November 2023, and subsequently by an online randomisation system called Sealed Envelope™. The randomisation system is stratified by site and minimised by age (65–79 vs 80 and over).

#### Concealment mechanism {16b}

The web-based and telephone randomisation systems ensure allocation concealment. Minimisation with probability weighting of 0.8 is used in order to reduce predictability. Participants are randomised once written consent has been provided and all baseline measurements completed.

#### Implementation {16c}

GCP-trained doctors, research nurses or delegated team members at the site enrol patients and use the automated randomisation system to assign the participants to the interventions. All patients that enter the study are given a unique, six-digit study (participant) identification number generated by the randomisation system.

### Assignment of interventions: Blinding

#### Who will be blinded {17a}

Due to the nature of the intervention, participants and those administering the intervention are not blinded to group allocation. Patients are, however, blinded to their randomised allocation prior to completion of their baseline questionnaire to remove bias. Two statisticians are supporting the trial. The senior statistician will remain blinded throughout the trial and be responsible for writing the statistical analysis plan (SAP) and attending TSC meetings. The other trial statistician will perform all disaggregated analyses according to a pre-specified statistical analysis plan and will attend closed Data Monitoring Committee (DMC) meetings, as required. The remaining members of the study team will remain blinded to aggregate data only.

#### Procedure for unblinding if needed {17b}

There is no requirement for unblinding.

### Data collection and management

#### Plans for assessment and collection of outcomes {18a}

##### Baseline assessment and data collection for the RCT

Following consent and prior to randomisation, participants complete a patient questionnaire and demographic, social, clinical, resource use and laboratory data (Table 1) are collected. The physical assessment is carried out by the research nurse following standard operating procedures. No additional blood or urine tests are required other than those already performed as part of routine care.

**Table 1:** Summary of baseline data collection for the RCT

Demographics/social	Age, sex, ethnicity, marital status, education level, distance lived from kidney clinic, alcohol consumption, smoking history
Clinical	Primary kidney disease, date first seen by nephrologist, comorbidities, dietary restrictions, prescribed medication
Resource use	Hospital/nursing home/residential home days/hospice days, other hospital outpatient services and primary care and community services in the last 4 months. Help from family, friends and carers in the last week
Laboratory	Creatinine, urea, albumin, haemoglobin, haematocrit, mean corpuscular volume, sodium, potassium, bicarbonate, corrected calcium, phosphate, intact parathyroid hormone, total cholesterol. (From the date of the study visit or the closest date prior to the study visit.)
Physical assessment	Height, weight, blood pressure, heart rate, waist circumference, timed get up and go [30], hand grip strength (Jamar hand dynamometer) [31], WHO performance status
Patient reported	EQ-5D-5L [25], IPOS-renal [26], ICECAP-O [27], ICECAP-SCM [28], MTBQ [29]
Relative/carer reported	PACKS impact on carers questionnaire [32]

##### Follow-up assessment and data collection for the RCT

Follow-up data collection for the RCT is summarised in Table 2. Clinical, resource use, laboratory and compliance with the intervention is collected by research nurses from primary and secondary care clinical notes and during study visits/contacts. The physical assessment is performed by the research nurse following standard operating procedures. No blood or urine tests are required other than those that will already have been performed as part of routine care.

**Table 2:** Summary of follow up data collection for the RCT

Clinical	Co-morbidities, hospital admissions including dates and causes, dialysis access surgery procedures and complications, other surgery, dialysis treatment received, dietary restrictions, prescribed medication, date, location and cause of death
Resource use	Hospital/nursing home/residential home days/hospice days, other hospital outpatient services and primary care and community services in the last 4 months. Help from family, friends and carers in the last week
Laboratory	Creatinine, urea (pre- and post-dialysis if on haemodialysis), albumin, haemoglobin, haematocrit, mean corpuscular volume, sodium, potassium, bicarbonate, corrected calcium, phosphate, intact parathyroid hormone, total cholesterol. (From the date of the study visit/contact or the closest date prior to the study visit/contact.)
Physical assessment	Weight, blood pressure, heart rate, waist circumference, timed get up and go [30], hand grip strength (Jamar hand dynamometer) [31], WHO performance status
Compliance with trial	Number of home visits by clinical team from kidney unit; Number of attendances at CKD clinic; Number of telephone contacts from clinical team at kidney unit; Number of visits from the palliative care team; Number of telephone contacts from the palliative care team; Advance care agreed; Advance care plan reviewed/updated; Cardiopulmonary resuscitation decision documented; Preferred place of death documented
Patient reported	EQ-5D-5L [25], IPOS-renal [26], ICECAP-O [27], ICECAP-SCM [28], MTBQ [29]
Relative/carer reported	PACKS impact on carers questionnaire [32], QUALYCARE post-bereavement survey [33]

Study visits/contacts are 4-monthly (+ / − 4 weeks) until withdrawal from the study, death or end of follow-up. They are arranged face-to-face once a year (at home or in clinic) and by telephone twice a year. Patient- and carer-reported outcomes are collected via postal or electronic questionnaires every 4 months, with carers also being asked to complete a proxy-report for the deceased’s quality of life in the last week of life.

##### Data linkage

At the point of consenting to take part in the RCT or the RFU, all participants are asked to consent to linkage to existing healthcare databases, such as Hospital Episode Statistics, the Office for National Statistics, and the UK Renal Registry, and their equivalents in devolved nations. This will provide data on dates and causes for all hospital admissions, date and cause of death, and dates and modalities of acute or chronic dialysis, if initiated. This will enable follow-up of outcomes (though not quality of life) for participants who wish to stop providing data to the study who might otherwise be lost to follow-up, including those who move to a kidney unit not participating in the trial.

##### Resource-use data collection

Data is collected as detailed in trial assessments and data collection. Costs from the NHS and PSS perspective will include costs associated with hospital, hospice and general practice and community care, and will include the costs of facilities, staff salaries and medication. For both interventions, appropriate preparation costs will be included, as will costs associated with delivery of the pathway of care experienced by the patient. Discussion with clinical collaborators prior to the start of the trial ensured that all relevant resource use is captured. For participants preparing for the responsive management pathway, this is likely to include additional support for the patient and their family/friends/carer, as well as routine monitoring. For participants preparing for dialysis, this will include all visits, scans, surgical appointments and surgical/radiological procedures. Where possible, resource-use data will be obtained routinely, through routine hospital data (e.g. hospital episode statistics), as such data are generally more comprehensive. Resource use that is not captured through routine sources is captured through the clinical trial documentation or questionnaires (adapted version of the Client Service Receipt Inventory) administered to patients and their family/friend/carer at follow-up as appropriate.

#### Plans to promote participant retention and complete follow-up {18b}

For patient-reported outcomes, up to three reminders are conducted. If a patient becomes too unwell to complete the patient questionnaires, a proxy report from a relative/carer is accepted. Patients who discontinue allocated treatment can choose to continue to receive patient questionnaires, attend follow-up contacts and allow data linkage.

#### Data management {19}

All data held in Bristol conforms to the University of Bristol Data Security Policy and is in compliance with the General Data Protection Regulation and the Data Protection Act 2018. Study data is collected and managed using REDCap [47] hosted at the University of Bristol. The database incorporates data entry and validation rules to reduce data entry errors, and management functions to facilitate auditing and data quality assurance.

Data collected on the paper CRFs at study centres, or as questionnaires from participants, is identifiable only by participant study number. Information capable of identifying individuals and the nature of treatment received is held in the database with restricted access to Prepare for Kidney Care study staff and essential national database staff. Some participants complete questionnaires online, and in such cases, these are completed directly onto a secure web-based database by the participants. Paperwork is transferred to the central study office via secure email or tamperproof envelopes and via recorded delivery.

Identifiable information, as agreed with partner organisations and set out in the participant information sheet, is used to link this primary dataset with existing routine healthcare databases for follow-up. The database system protects patient information in line with the data protection legislation and any specific requirements of the partner organisations. Trial and database staff ensure that participants’ anonymity is maintained through protective and secure handling and storage of patient information. The chief investigator acts as custodian of the full dataset.

##### Retention of data

Completed case report forms will be kept for 5 years following the end of the study to enable audit of data used in publications. These will be kept at the UoB for this time and then destroyed.

#### Confidentiality {27}

Personal identifiable and clinical data will be processed in compliance with the Common Law Duty of Confidentiality and Data Protection Act 2018, as set out in the privacy notice on the trial website [21].

#### Plans for collection, laboratory evaluation and storage of biological specimens for genetic or molecular analysis in this trial/future use {33}

No biological specimens are being collected.

## Statistical methods

### Statistical methods for primary and secondary outcomes {20a}

All data analysis will be in accordance with the Consolidating Standards of Reporting Trials (CONSORT) [48, 49] guidelines and the primary statistical analyses will be conducted on a modified intention-to-treat basis including all participants contributing at least one quality of life measurement. Baseline variables to be explored are shown (Table 1). Patient-reported outcome scores based on standardised questionnaires will be calculated based on the developers’ scoring manuals and missing and erroneous items will be handled according to these manuals. Continuous measures will be presented as means and standard deviations or medians and ranges depending on their distribution. Categorical data will be presented as frequencies and proportions. A full statistical analysis plan will be developed and agreed by the Trial Steering Committee (TSC) prior to undertaking any analyses of the trial data.

#### Primary outcome analysis

The primary endpoint in this study is quality-adjusted life years which will be calculated using EQ-5D-5L data collected 4-monthly and survival data. The primary statistical analyses between the randomised groups will be conducted on a modified intention-to-treat basis including all participants contributing at least one quality of life measurement. Utility scores will be estimated from the EQ-5D-5L using the NICE-recommended approach at the time of analysis and these will be used to compute QALYs experienced over the follow-up period using the area under the curve approach. Depending on the distribution of the observed QALYs data, appropriate regression techniques will be applied. If normally distributed, the mean differences in QALYs between treatment arms with 95% confidence intervals will be calculated using multivariate linear regression models. Otherwise, the shape of the distribution will be considered to inform the choice of alternative, suitable regression models (including non-parametric models) for this outcome. All models will adjust for baseline EQ-5D-5L scores.

#### Secondary outcome analysis

Survival will be assessed using Kaplan–Meier curves and a Cox proportional hazard model analysis. Hospital-free days will be studied using appropriate regression models—such as the negative binomial model with offset for the duration of follow-up—based on the distribution of the data. Appropriate repeated measures regression models for patient-reported outcome scores will be chosen based on the distribution of each outcome.

A detailed statistical analysis plan will be developed for the approval of the TSC prior to analysis. Any deviations from the approved plan will be described and justified to the TSC for their approval.

#### Economic data analysis

The trial includes a formal economic evaluation comparing the costs and outcomes of the ‘preparing for responsive management’ and ‘preparing for renal dialysis’ arms of the trial from the perspectives of (a) the NHS and PSS and (b) society, including patients and close persons, from the point of randomisation (i) up to an estimated average of 3 years based on trial data alone and (ii) to death based on trial data combined with decision modelling. Economic evaluation will take the form of (i) cost-effectiveness analysis using QALYs generated using EQ-5D-5L, (ii) cost-effectiveness analysis using years of full/sufficient capability equivalence gained using ICECAP, (iii) cost consequences analysis including all costs and outcomes presented in disaggregated format. QALYs will be estimated from EQ-5D-5L scores as specified in the “Primary outcome analysis”.

Valuations will be assigned to capability outcomes based on published UK population tariffs at the end of the study [27]. All resource use will be valued using unit costs derived from national sources where available.

The economic data analysis will be conducted on an intention-to-treat basis using an incremental approach. Mean total costs and outcomes will be calculated across all patients and incremental cost-effectiveness ratios for the trial arms will be estimated to produce an incremental cost per QALY gained/cost per year of full/sufficient capability equivalent gained from both health and societal perspectives. Missing cost and outcome data will be imputed using appropriate methods, if appropriate [50]. To avoid bias, imbalances in baseline utility/capability/costs between the groups will be controlled for [51]. Discounting will be applied at the NICE-recommended rate of 3.5% [52]. Given the multicentre nature of this study, it may be appropriate to use hierarchical modelling techniques (with explanatory variables stratified into patient and centre levels).

It is anticipated that significant numbers of patients may not have died by the end of the trial and so modelling will need to be used to extrapolate beyond the trial, to capture costs and impacts for the remaining lifetime. Markov models will be developed to simulate the clinical pathways of patients with advanced CKD for both economic outcomes. Markov models are appropriate as they can represent situations where patients change from one state to another (for example, the shift to dialysis) as well as experiencing recurrent states (such as remaining on dialysis) over long periods of time. The models will be structured such that clinical pathways are based on the arms of the trial. Transition probabilities, costs and outcome information (in terms of both health-related QoL and capability wellbeing) will be taken primarily from the trial, but will be supplemented with information from routine datasets and published data, where necessary; time-dependent probabilities will be used as in other Markov models of advanced CKD [53]. Time intervals for transitions between states will be based on the four-monthly follow-up period within the trial.

Uncertainty will be explored using deterministic (trial and model) and probabilistic (model only) sensitivity analyses. Deterministic sensitivity analysis will focus particularly on those issues where it has been necessary to make assumptions about resource use and/or cost or where particular issues have been encountered. Probabilistic sensitivity analysis will be used to estimate the joint effect of uncertainty in the model parameters. Results will be presented as the incremental cost-effectiveness ratio and using cost-effectiveness acceptability curves to show the probability that the results fall below given cost-effectiveness thresholds. Results will be compared to the NICE recommended cost per QALY threshold at the time of the completion of the study [52] Thresholds for the ICECAP measures will be applied based on deliberative research for ICECAP-A [54].

#### QRI and qualitative data analysis

Analysis of QRI data.

Screening log data will be analysed and summarised descriptively. Interviews will be audio-recorded using digital encrypted recorders, transcribed verbatim and edited to ensure anonymity and managed using NVivo software (QRS International). Transcripts will be analysed thematically using constant comparative approaches adopted from Grounded Theory [55]. Audio-recorded recruitment appointments will be subjected to content, thematic and novel analytical approaches, including targeted conversation analysis [56]. Standard approaches to enhancing rigour, such as double-coding and seeking out ‘negative cases’, will be employed throughout the conduct of the QRI. Descriptive accounts of each qualitative data source will be written and iteratively updated as data collection and analysis progress, enabling ongoing discussion and joint interpretation of data amongst the QRI team. A detailed description of approaches to analysing and triangulating QRI data is published elsewhere [46].

Analysis of interviews investigating acceptability and compliance issues.

Interviews conducted with HCPs and trial participants/relatives will be analysed thematically, as reported above, with the same approaches to safeguarding rigour employed throughout (e.g. double coding, production of iterative descriptive accounts, and regular discussion of evolving findings in team meetings). Repeat interviews for trial participants will be analysed and written up as individual case studies, with cross case-comparisons informing overarching findings from this group as data collection/analysis progresses. We will code and write up findings from each stakeholder group (i.e. patients, relatives, HCPs) separately in the early stages of analysis, with regular cross-checking of themes emerging from each group to inform new lines of enquiry and promote interrogation of the data through different lenses. With time, findings from each group will be triangulated to inform a single report.

### Interim analyses {21b}

No formal interim statistical analyses of the principal outcome measure are planned. However, mortality rates will be reported routinely to the DMC by arm (unblinded). The DMC will consider a recommendation to discontinue recruitment, in all patients or in selected subgroups, only if the result is likely to convince a broad range of clinicians, including those supporting the trial and the general clinical community.

### Methods for additional analyses (e.g. subgroup analyses) {20b}

We will conduct pre-planned subgroup analyses to investigate any differential effects according to a number of factors. These will be done by introducing appropriate interaction terms in the regression models. We will carry out these analyses by age at baseline (65–79 years vs ≥ 80 years) and rate of kidney function decline in the 12 months pre-baseline (≤ 5 ml/min/1.73 m^2^ vs > 5 ml/min/1.73 m^2^). In the group aged 80 years and over we will also stratify according to comorbidity scores at baseline (< 2 vs ≥ 2) and WHO performance status at baseline (< 3 vs ≥ 3).

### Methods in analysis to handle protocol non-adherence and any statistical methods to handle missing data {20c}

Where missing data exist, the frequency of missing data will be indicated and if the amount of missing data differs substantially between treatment groups (> 10%) potential reasons will be explored. Sensitivity analyses will be conducted (including the use of multiple imputation methods where assumptions are met) to examine the influence of missing data on the key trial findings.

### Plans to give access to the full protocol, participant-level data and statistical code {31c}

The full protocol is available as a supplement. External groups will be able to apply to the trial management group to request access to anonymised participant-level data, as permitted by the data sharing agreements for data that has been provided through linkage.

## Oversight and monitoring

### Composition of the coordinating centre and trial steering committee {5d}

The study is supervised by a TMG consisting of grant holders and other relevant trial delivery staff. A core delivery team at the coordinating centre, consisting of chief investigator, trial manager, trial administrator and lead research nurse, meet monthly. The full TMG meets 6–12 monthly. A TSC oversees the progress of the trial and comprises of an independent chair and five other independent members, including a public and patient involvement (PPI) representative and the CI. A PAG also oversees the progress of the trial and advises on elements of trial design and conduct from the patient and public perspective. The chair of the PAG also sits on the TMG and the TSC has a PPI co-chair. The Sponsor is responsible for overall oversight of the trial.

### Composition of the data monitoring committee, its role and reporting structure {21a}

A DMC monitors accumulating trial data for quality, completeness and patient safety and comprises of an independent chair and at least two other independent members. The CI, trial manager and senior statistician attend open meetings of the DMC; one designated statistician from the coordinating centre prepares reports for, and attends, closed meetings of the DMC. The DMC meets 6–12 monthly, 2–4 weeks before the TSC meets, and provides a report to the chair of the TSC for consideration at that meeting. The DMC charter is available from the corresponding author.

### Adverse event reporting and harms {22}

Given the intensive monitoring of patients with advanced CKD in routine clinical care, the comprehensive data on clinical events recorded directly by the trials unit, and the routine use of pathways that involve preparing for dialysis and preparing for conservative care in the NHS, the study utilises the following risk-adapted safety reporting approach:

1. Serious adverse events are collected as part of the study data on the trial CRFs, including an assessment of expectedness and relatedness by the site.
2. Only SAEs categorised as unexpected and causally related to the intervention require expedited reporting to the CI and sponsor.

Deaths, quality of life and SAEs categorised as unexpected and causally related to the intervention are regularly reviewed by the DMC.

### Frequency and plans for auditing trial conduct {23}

The study is monitored in accordance with North Bristol NHS Trust’s Monitoring standard operating procedure. All trial-related documents will be made available on request for monitoring and audit by North Bristol NHS Trust, the Research Ethics Committee and other licensed bodies. Monitoring and audits undertaken by North Bristol NHS Trust, under their remit as sponsor, or individuals appointed responsibility for monitoring on behalf of the Trust, will ensure adherence to GCP and the NHS Research Governance Framework for Health and Social Care (2nd edition). Remote monitoring is conducted based on information submitted by sites and analysis of the trial database. Site visits are initiated using a risk-based approach.

### Plans for communicating important protocol amendments to relevant parties (e.g. trial participants, ethical committees) {25}

All changes to the protocol will seek approval from the Sponsor, Research Ethics Committee, Health Research Authority and site research and development offices before local implementation. As judged necessary by the Sponsor and Research Ethics Committee, these changes will be communicated to the participants.

### Dissemination plans {31a}

The results of the study will be published in the academic press and presented at national and international conferences. The investigators will work with guideline writing organisations to ensure that they are aware of the new data and encouraged to incorporate these data into updates of their guidance. Working with the PAG, the investigators will develop plain English summaries of the results of the trial for sharing with trial participants and disseminating to patients and the public more widely.

## Discussion

People with advanced CKD urgently need unbiased information to inform decisions about whether to start dialysis or continue with holistic patient-centred care without dialysis. While the risk of bias in observational studies addressing this issue is recognised to be high, the possibility of conducting an RCT comparing dialysis and conservative kidney management pathways was until recently considered too difficult and possibly unethical [57]. Preparatory work provided evidence that challenged this, however, most notably a survey of 41 kidney units in the UK which reported that the percentage of people aged 75 years and over choosing not to have dialysis varied from less than 10% in seven kidney units to more than 80% in six kidney units [6]. This simple statistic proved very powerful in convincing clinicians, funders and the research ethics committee that there was uncertainty (or equipoise) about the ‘right’ treatment within the kidney community, if not at the individual clinician level.

Another more nuanced, but fundamental, concern was whether the RCT approach was the right one for such a complex treatment decision, which in routine practice would be made with knowledge of the individual patient’s priorities for care and awareness of the strengths of the local service. This issue speaks to a wider concern often voiced that RCTs tend to be positivist—reporting average effects across a population rather than exploring the different effects a treatment might have depending on the context in which it is delivered [58]. In Prepare for Kidney Care, we have tried to incorporate both a positivist approach (with a primary outcome measure based on difference in QALYs between the two arms) and a realist approach (with an underpinning theoretical framework, embedded mixed methods to understand experiences, and multiple secondary outcome measures and planned sub-group analyses) [59].

Related to this is the concern that the recruited participants will be highly selected, and it will be difficult to know how the results of the RCT can be applied to all patients with advanced CKD meeting the eligibility criteria. Crucial to addressing this is the embedding of the RCT in both the registry follow-up study (which includes patients attending kidney units who meet the eligibility criteria but are unable to agree to randomisation) and the routine data study (which includes all patients in kidney units who meet the eligibility criteria, regardless of whether they have been screened or approached to participate). This latter approach utilises novel data capture by the UK Renal Registry, with permission to collect data for quality assurance and research on all patients attending kidney units with an eGFR less than 90 mL/min/1.73 m^2^ [60], and will allow the generalisability of the RCT results to be understood.

Finally, it has always been recognised that recruitment to the RCT would be challenging. Instrumental in addressing this concern was experience from the QRI team, whose mixed methods approach had contributed to the successful recruitment of patients to a number of challenging trials, mainly surgical [44]. Amongst other things, this embedded approach allowed the rapid sharing of best practice in recruitment conversations and highlighted novel challenges to recruitment. A major challenge has been the chronic nature of the treatment decision to follow a prepare for dialysis or conservative kidney management pathway; one which is likely to change as a patient’s life changes. Beyond its primary purpose of optimising the recruitment and consent process, the QRI work will therefore contribute to a realist approach to the evaluation of the intervention, provide wider lessons on recruitment to challenging non-surgical trials, and provide lessons on communication in shared decision making in advanced CKD in routine clinical practice.

## Trial status

Recruitment commenced in July 2017 and is scheduled to complete by end of August 2024. Follow-up is expected to continue to end of August 2025. The current protocol is version 10.0, dated 26 June 2024.

## Supplementary Information

Supplementary Material 1.

Supplementary Material 2.

Supplementary Material 3.

## Data Availability

The University of Bristol will hold the final dataset. External groups will be able to apply to the TMG to request access to anonymised patient level data, as permitted by the data sharing agreements for data that has been provided through linkage.

## Abbreviations

BTC: Bristol Trials Centre
CI: Chief Investigator
CKD: Chronic kidney disease
CRF: Case Report Form
DMC: Data Monitoring Committee
eGFR: Estimated glomerular filtration rate
EQ-5D-5L: EuroQol 5-dimension 5-level
GCP: Good Clinical Practice
GP: General practitioner
HD: Haemodialysis
HR: Hazard ratio
ICECAP-O: ICEpop CAPability for older people
ICECAP-SCM: ICEpop CAPability supportive care measure
IPOS-renal: Integrated palliative outcome scale-renal
ISRCTN: International Standard Randomised Controlled Trials Number
NHS: National Health Service
NICE: National Institute for Health and Care Excellence
PACKS: PAlliative Care in chronic Kidney diSease impact on carers
PAG: Patient Advisory Group
PSS: Personal Social Services
PI: Principal Investigator
QALY: Quality-adjusted life year
QoL: Quality of life
QRI: QuinteT Recruitment Intervention
RCT: Randomised control trial
REC: Research Ethics Committee
RFU: Registry Follow Up study
TMG: Trial Management Group
TSC: Trial Steering Committee
UK: United Kingdom
UKRR: United Kingdom Renal Registry
WHO: World Health Organization
