# Supplementary material for: Preparing for responsive management versus preparing for renal dialysis in multimorbid older people with advanced chronic kidney disease (Prepare for Kidney Care): Study protocol for a randomised controlled trial
Source: Trials. 2024 Oct 17;25:688. doi: 10.1186/s13063-024-08509-8 (PMC11487988; doi:10.1186/s13063-024-08509-8)
Supplement: Supplementary file 1 — Supplementary Material 1. [file 13063_2024_8509_MOESM1_ESM.docx]

# **Additional file 1 - Registry Follow Up study**

Eligible patients who decline participation in the RCT are documented in screening logs and invited to consent to participate in a cohort study. The overall aim of this study is to assess the external validity of the randomised controlled trial by comparing baseline characteristics and outcomes of two groups of patients:

1. Participants in the Prepare for Kidney Care RCT
2. Non-participants in the Prepare for Kidney Care study RCT who consent for registry follow-up (the focus of this appendix)

In addition, using routine NHS data, a third cohort will be created: people aged 65 or older with an eGFR less than 15mL/min/1.73m^2^ not included in 1) or 2). If the data allows, this cohort will be further restricted to people with two or more co-morbidities to more closely mimic the RCT. As these patients are not being asked to give individual consent for their data to be analysed, appropriate applications will be made to the Health Research Authority for Section 251 support, and equivalents, to carry out this comparison.

**Data collection for the Registry Follow Up study**

**Baseline data**

Demographic, social, clinical, resource use, laboratory and patient/ carer reported data is collected by research nurses during study visits at baseline (following consent). The physical assessment is performed by the research nurse following standard operating procedures. No blood or urine tests are required other than those that will already have been performed as part of routine care.

**Table 1 Summary of baseline data for the registry follow up study.**

| Demographics/ social | Age, sex, ethnicity, marital status, education level, distance lived from kidney clinic, alcohol consumption, smoking history |
| --- | --- |
| Clinical | Primary kidney disease, date first seen by nephrologist, co-morbidities, dietary restrictions, prescribed medication |
| Laboratory | Creatinine, urea, albumin, haemoglobin, haematocrit, mean corpuscular volume, sodium, potassium, bicarbonate, corrected calcium, phosphate, intact parathyroid hormone, total cholesterol. (From the date of the study visit or the closest date prior to the study visit.) |
| Physical assessment | Height, weight, blood pressure, heart rate, waist circumference, timed get up and go (1), hand grip strength (Jamar hand dynamometer) (2), WHO performance status. |
| Patient reported | EQ-5D-5L (3), IPOS-renal (4), ICECAP-O (5), ICECAP-SCM (6), MTBQ (7) |

**Follow up data**

For Registry Follow Up participants there are no study visits following the baseline study visit. All participants are asked at recruitment to consent to researchers having access to their primary and secondary care clinical notes and to linkage to existing healthcare databases, such as Hospital Episode Statistics, the Office for National Statistics and the UK Renal Registry. This will provide outcomes data such as commencement of acute or chronic dialysis, hospital outpatient attendances, hospital admissions for medical and surgical reasons and date and cause of death, including for participants that might otherwise be lost to follow up, for example if they move to a non-participating renal unit.

Once a year, participants are sent a patient questionnaire for completion and return by post or electronically. This will continue for the duration of the RCT follow up.

**Statistical analyses**

We will use descriptive statistics to describe the baseline characteristics of (1) participants in the Prepare for Kidney Care RCT, (2) participants in the Registry Follow Up study, and (3) people identified as eligible for the RCT in routine NHS data. This will include age, gender, social deprivation, rate of decline in eGFR in the past 12 months, co-morbidity and dialysis decision making status. For those patients participating in the RCT and participating in the Registry Follow Up study, we will also describe differences in quality of life and symptom burden at baseline.

We will use descriptive statistics to describe the outcomes of the three cohorts in terms of rate of decline in eGFR, rate of decline in quality of life, rate of increase in symptoms. We will describe hospital admissions rates, dialysis initiation rates, and survival. In the Registry Follow Up study, participants will be groups according to kidney failure treatment plan at baseline (i.e., dialysis, conservative kidney management, or undecided) and the kidney failure treatment received.

A full Statistical Analysis Plan will be developed and reviewed by the Trial Steering Committee.

## **Table 2. Schedule of Procedures for Registry Follow Up study**

| **Procedures** | **Screening** | **Baseline** | **Follow Up** |
| --- | --- | --- | --- |
|  | Face-to-face visits  -3, -2, -1 (as required) | Face-to-face visit 1 | (Linkage and questionnaire) |
| Eligibility assessment | √ |  |  |
| Informed consent | √ |  |  |
| Demographics |  | √ |  |
| Medical history |  | √ | √* |
| Medications |  | √ |  |
| Laboratory tests |  | √ | √** |
| Height |  | √ |  |
| Weight |  | √ |  |
| Blood pressure |  | √ |  |
| Heart rate |  | √ |  |
| WHO Performance Status |  | √ |  |
| Patient questionnaires*** |  | √ | √ |

* Including hospital outpatient visits, hospital admissions, dialysis initiation, and date and cause of death

** Where available from the UK Renal Registry

*** EQ-5D-5L (3), IPOS-renal (4), ICECAP-O (5), ICECAP-SCM (6), MTBQ (7)

**References**

1. Podsiadlo D, Richardson S. The timed "Up & Go": a test of basic functional mobility for frail elderly persons. Journal of the American Geriatrics Society. 1991;39(2):142-8.

2. Roberts HC, Denison HJ, Martin HJ, Patel HP, Syddall H, Cooper C, et al. A review of the measurement of grip strength in clinical and epidemiological studies: towards a standardised approach. Age and ageing. 2011;40(4):423-9.

3. Oppe M, Devlin NJ, van Hout B, Krabbe PFM, de Charro F. A Program of Methodological Research to Arrive at the New International EQ-5D-5L Valuation Protocol. Value in Health. 2014;17(4):445-53.

4. Murphy EL, Murtagh FEM, Carey I, Sheerin NS. Understanding Symptoms in Patients with Advanced Chronic Kidney Disease Managed without Dialysis: Use of a Short Patient-Completed Assessment Tool. Nephron Clin Pract. 2009;111(1):C74-C80.

5. Coast J, Flynn TN, Natarajan L, Sproston K, Lewis J, Louviere JJ, et al. Valuing the ICECAP capability index for older people. Social science & medicine. 2008;67(5):874-82.

6. Sutton EJ, Coast J. Development of a supportive care measure for economic evaluation of end-of-life care using qualitative methods. Palliative medicine. 2014;28(2):151-7.

7. Man MS, Chaplin K, Mann C, Bower P, Brookes S, Fitzpatrick B, et al. Improving the management of multimorbidity in general practice: protocol of a cluster randomised controlled trial (The 3D Study). BMJ open. 2016;6(4):e011261.
