# Supplementary material for: Preparing for responsive management versus preparing for renal dialysis in multimorbid older people with advanced chronic kidney disease (Prepare for Kidney Care): Study protocol for a randomised controlled trial
Source: Trials. 2024 Oct 17;25:688. doi: 10.1186/s13063-024-08509-8 (PMC11487988; doi:10.1186/s13063-024-08509-8)
Supplement: Supplementary file 2 — Supplementary Material 2. [file 13063_2024_8509_MOESM2_ESM.docx]

# **Additional File 2 – Stage 3 qualitative**

**Qualitative investigation to understand acceptability of trial arms/processes and reasons for non-compliance (stage 3 of the embedded qualitative/mixed-methods research).**

*Aim:*

Stage 3 of the integrated qualitative/mixed-methods research aims to understand the acceptability of the trial interventions and reasons for non-compliance/protocol deviations, from the perspectives of patients, relatives and the health and care professionals (HCPs) involved in patient care.

*Overview of methods:*

Semi-structured interviews will be conducted with three groups: trial participants, their family members/carers, and HCPs involved in their care.

There will be an intention to interview trial participants (and/or family members/ friends/ carers) within the first year of randomisation, with follow up interviews arranged at regular (but flexible) intervals to construct longitudinal summaries of their experiences over time. The frequency and timing of subsequent interviews will vary depending on clinical events and participant preferences, but it is anticipated that at least one follow up interview will be conducted each year. In addition to the above, patients who decide to discontinue trial treatment and withdraw from the RCT (and/or their family members/ friends/ carers) will be invited to take part in a specific interview that explores the reasons underlying these events.

Health and care professionals involved in patient care/decision making around treatment post randomisation will also be approached for interview. This will include, but not be limited to, nurses, GPs, renal consultants, and palliative care specialists.

Where possible, interviews will be supported with observational data of how the protocol is being implemented in practice, where possible. Nurse follow-up visits and telephone/ video discussions with patients will be observed and recorded (with informed consent being obtained) to understand the content and nature of these appointments and any difficulties in implementing the intervention or trial processes.

In addition to the above, the acceptability of trial processes and end-of-life care will be specifically explored with family members/ friends/ carers of deceased patients.

*Sampling:*

All patients who consent to taking part in the trial will be eligible for the stage 3 qualitative study.

Sampling will be purposeful, primarily with an intention to capture experiences of participants from a range of sites, with attention paid to gender and age/comorbidity status on trial entry (i.e. key elements of the eligibility criteria). We will prioritise sampling patients allocated to the intervention, but include individuals allocated to renal dialysis for comparative purposes. Sampling may also be driven be emerging findings, where new lines of enquiry emerge based on concurrent analysis.

Health and care professionals will be sampled on a key informant basis, based on their role in delivering care to patients post-randomisation. We will focus primarily on staff involved in delivering responsive management, but include individuals who specialise in delivering dialysis for comparative purposes. As above, sampling will likely be driven by emerging findings for this group, where insights from concurrent analysis raise new lines of enquiry that are best addressed by approaching particular key informants with relevant knowledge/expertise.

*Data collection*

Semi-structured interviews will be guided via topic guides that will evolve as data collection proceeds. Distinct topic guides will be used for each group and type of interview (e.g. repeat interviews will be distinct to initial interviews).

A trained qualitative methodologist will conduct all interviews, either face to face or via telephone or web-conferencing software, following receipt of written or verbal informed consent. All interviews will be audio-recorded on encrypted digital devices.
