# Supplementary material for: Preparing for responsive management versus preparing for renal dialysis in multimorbid older people with advanced chronic kidney disease (Prepare for Kidney Care): Study protocol for a randomised controlled trial
Source: Trials. 2024 Oct 17;25:688. doi: 10.1186/s13063-024-08509-8 (PMC11487988; doi:10.1186/s13063-024-08509-8)
Supplement: Supplementary file 3 — Supplementary Material 3. [file 13063_2024_8509_MOESM3_ESM.docx]

# **Additional File 3 - QuinteT Recruitment Intervention (QRI)**

The QRI will proceed as two iterative phases, as outlined below.

**Phase I: understanding recruitment issues**

Phase I will focus on building up a comprehensive understanding of recruitment challenges that arise as sites open to recruitment. A multi-faceted, flexible approach will be adopted, using one or more of the following methods:

1. **Semi-structured Interviews:** Semi-structured interviews will be conducted with one or more of the following groups:
   1. Members of the TMG, including the Chief Investigator and those closely involved in the design, management, leadership, and coordination of the trial.
   2. Site personnel with a role in shaping recruitment processes (referred to as ‘site staff’).
   3. Eligible patients who are approached to take part in the RCT.

Interviews with TMG members/site staff will investigate their perspectives on the RCT and experiences of recruitment processes (where relevant). Key topics explored will include views about the trial design and protocol; interpretations of the evidence on which the trial is based; perceptions of equipoise; methods for identifying eligible patients and views on the eligibility criteria, and examples of actual recruitment successes and difficulties. Sampling will proceed on a key informed basis, with attempts to achieve representation from all sites. Interviews with patients, if conducted, will explore views on the presentation of study information, interpretations of trial processes (e.g. randomisation), and reasons underlying decisions to accept or decline the trial. Patients will be purposefully selected, to build a sample of maximum variation based on age, multi-morbidity status, gender, study site, and the final decision about trial participation (i.e. accept or decline).

Numbers of interviews conducted with each of the above groups will be guided by the concept of data saturation and pragmatic considerations (e.g. all key informants in a particular site have been approached).

QRI interviews will take place at a mutually convenient location, in a suitably private and quiet setting. All participants will be offered the option to conduct the interview over the telephone or a secure audio-visual platform. The University of Bristol’s ‘lone researcher’ safety policies will be upheld for any interviews taking place in non-public settings (e.g. participants’ homes).

1. **Screening log analyses and mapping recruitment pathways:** The QRI team will work closely with the Clinical Trials Unit (CTU) to design a screening log that captures the numbers of patients screened, eligible, approached, randomised (‘SEAR’), and numbers that accept/decline their allocation, based on the QuinteT SEAR template (1). The SEAR template also prompts sites to record reasons why patients were ineligible, not approached, not randomised, or declined their allocation. In collaboration with the CTU, the QRI team will produce regular summaries of descriptive analyses of SEAR data, to help identify points at which patients do not continue with recruitment to the RCT. The interviews with recruiters (above) will be used to map out the recruitment pathway for each site, noting processes for screening and identifying eligible patients, how patients are approached, and the personnel involved in these activities. Recruitment pathways will be compared with screening log data to identify ‘bottlenecks’ in the recruitment pathway for each site and practices that are conductive or counter-productive to efficient and effective recruitment.
2. **Audio recording and observation of recruitment appointments:** Scheduled appointments and/or home visits during which the trial is discussed will be audio-recorded and/or observed with permission, including conversations carried out remotely. All staff involved in discussing Prepare for Kidney Care with patients will be invited to record their discussions with patients using an encrypted platform of choice. We will pay particular attention to: whether the trial interventions are described in a clear, accurate and balanced way; management of patient preferences; and explanations of trial processes (e.g. randomisation, follow-up). Recordings will be transferred to and from the University of Bristol (for analysis) through University of Bristol-approved secure data transfer facilities and/or encrypted flash drives that adhere to NHS Trust policies.
3. **Observation of TMG and investigator meetings:** It is likely that the CI, TMG and clinical investigators will meet or have telephone/ video conferences to discuss the progress of the RCT. The QRI team will observe and potentially record these meetings, with permission. The aim will be to gather further information about specific issues that may have a bearing on recruitment. These meetings can also elucidate new solutions to recruitment difficulties.
4. **Document analysis of trial materials:** Patient Information Sheets (PISs), the study protocol, and other patient/public-facing information about the trial will be scrutinised to identify aspects that are unclear or potentially open to misinterpretation, thus having a possible bearing on recruitment.

Findings from the above sources will be triangulated, to develop a rapid and in-depth understanding of factors shaping recruitment to Prepare for Kidney Care, which will be iteratively shared with the Chief investigator/TMG.

**Phase 2: Development and implementation of recruitment strategies**

The QRI team will work closely with the TMG to design and implement tailored actions to support recruitment, informed by Phase 1 findings. Actions may be applicable to all sites, specific sites, or individual recruiters, examples of which include:

1. Disseminating ‘tips’ documents with suggestions on how to explain the trial;
2. Refinements to patient-facing materials (e.g. to address common misconceptions)
3. Group ‘feedback sessions’, to tackle issues relating to eligibility assessments/ perceptions of equipoise, and communication issues highlighted by audio-recorded consultation data.
4. Site-visits to discuss strategies for improving how recruitment is organised and delivered, facilitated by sharing examples of successful recruitment models from other sites.
5. Individual confidential feedback to recruiters who provide recordings of their consultations.

**Iterative nature of Phases 1 and 2**

The QRI has been presented as two distinct phases for clarity, but these phases are likely to overlap or run in tandem in practice. For instance, new avenues of enquiry may emerge through feedback meetings, which can be a route to investigating recruitment issues in their own right.

The QRI will continue to investigate how recruitment proceeds following delivering of ‘actions’, to identify the need for further intervention and/or identify new issues that arise. As new sites open to recruitment, they will be provided with tailored ‘pre-emptive’ recruitment training and support, informed by QRI findings to date.

**References**

1. Wilson C, Rooshenas L, Paramasivan S, Elliott D, Jepson M, Strong S, et al. Development of a framework to improve the process of recruitment to randomised controlled trials (RCTs): the SEAR (Screened, Eligible, Approached, Randomised) framework. Trials. 2018;19(1):50.
